# The predictive significance of CD20 expression in B-cell lymphomas

**DOI:** 10.1186/1746-1596-6-33

**Published:** 2011-04-12

**Authors:** Veronika Kloboves Prevodnik, Jaka Lavrenčak, Mateja Horvat, Barbara Jezeršek Novakovič

**Affiliations:** 1 Institute of Oncology, Department of Cytopathology, Ljubljana, Slovenia; 2 Bayer d.o.o., Ljubljana, Slovenia; 3 Institute of Oncology, Department of Medical Oncology, Ljubljana, Slovenia

## Abstract

**Background:**

In our recent study, we determined the cut-off value of CD20 expression at the level of 25 000 molecules of equivalent soluble fluorochrome (MESF) to be the predictor of response to rituximab containing treatment in patients with B-cell lymphomas. In 17.5% of patients, who had the level of CD20 expression below the cut-off value, the response to rituximab containing treatment was significantly worse than in the rest of the patients with the level of CD20 expression above the cut-off value. The proportion of patients with low CD20 expression who might not benefit from rituximab containing treatment was not necessarily representative. Therefore the aim of this study was to quantify the CD20 expression in a larger series of patients with B-cell lymphomas which might allow us to determine more reliably the proportion of patients with the CD20 expression below the cut-off.

**Methods:**

Cytological samples of 64 diffuse large B-cell lymphomas (DLBCL), 56 follicular lymphomas (FL), 31 chronic lymphocytic leukemias (CLL), 34 mantle cell lymphomas (MCL), 18 marginal zone lymphomas (MZL) and 15 B-cell lymphomas unclassified were analyzed for CD20 expression by quantitative four-color flow cytometric measurements using FACSCalibur flow cytometer (BD Biosciences).

**Results:**

The range of CD20 expression in different B-cell lymphomas was very broad, varying from 2 737 to 115 623 MESF in CLL and 3 549 to 679 577 MESF in DLBCL. However, when we compared the CD20 expression in the groups of patients with DLBCL, FL, MCL, MZL, CLL and B-cell lymphomas unclassified, it was found to be significantly lower (p = 0.002) only in CLL but did not significantly differ in other lymphoma types (p = NS). Fifty-three out of 218 (24.3%) patients with B-cell lymphomas had the CD20 expression below the cut-off value.

**Conclusions:**

The CD20 expression in CLL is significantly lower than in most histological types of mature B-cell lymphomas in which it appears to be comparable. Approximately 25% of B-cell lymphoma patients have the CD20 expression below the cut-off value showing that the low CD20 expression might be more common than presumed from our previous study.

## Background

The study of CD20 expression (cluster of differentiation antigens) in lymphoma cells is vital not only to establish an accurate diagnosis but also to prepare an appropriate plan of treatment with biological drugs [1,2]. As rituximab is a biological drug most frequently used in the treatment of B-cell lymphomas, the study of CD20 expression is becoming increasingly important, in particular in the light of most effective treatment planning and appropriate following-up of response to treatment. Even so, only few studies on CD20 expression in the patients with different histology types of B-cell lymphoma have been so far performed. In these few studies, it has been confirmed that, in the majority of B-cell lymphomas, the CD20 antigen is expressed on the surface of neoplastic cells; however, the intensity of CD20 expression varies by the type of lymphoma and by the differentiation of lymphoma B-cells. It is therefore assumed that the CD20 expression is low in the cells of B-cell chronic lymphocytic leukemia (CLL), while it is most intense in the cells of diffuse large B-cell lymphoma (DLBCL) and of hairy cell leukemia [3-5]; by contrast, it is only exceptionally aberrantly expressed in the cells of multiple myeloma [6,7].

In addition, only sporadic studies have been investigating whether the level of CD20 expression is related to the response to treatment with rituximab. The results of two studies concluded recently showed indeed that the clinical outcome of patients with low CD20 expression was worse than that of patients with high CD20 expression [8,9]. One of these studies was performed in our laboratory. In this study, we determined the cut-off value of CD20 expression at the level of 25 000 molecules of equivalent soluble fluorochrome (MESF) to be the predictor of response to rituximab containing treatment in the patients with B-cell lymphomas. Namely, the patients with low CD20 expression responded to rituxmab containing treatment poorly or not at all compared to patients with the CD20 expression above the cut-off level. We also observed that slightly less than one fifth of patients (17.5%) with B-cell lymphomas had the CD20 expression below the cut-off value [9].

The purposes of the present study were firstly to assess the level of CD20 expression in B-cell lymphomas most frequently occurring in Slovenia where the incidence of B-cell lymphoma is similar to that in Western Europe and North America [10,11] and secondly to determine the proportion of patients with the CD20 expression below the cut-off value of 25 000 MESF in a larger series of patients.

## Methods

Two hundred and eighteen patients diagnosed and treated for primary or recurrent lymphoma at the Institute of Oncology Ljubljana, Slovenia between 2003 and 2007 were included in the study. By the time of cytological sampling, none of the patients had been treated with rituximab. In all patients, the primary or recurrent lymphoma had been suspected clinically. Cytological examination with flow cytometric immunophenotyping (FCI) was performed to make or confirm the diagnosis of lymphoma. The cytological diagnosis was further verified by excisional biopsy and histological examination in most cases. Only exceptionally, when the excisional biopsy could not be performed, the cytological diagnosis was validated through clinical follow-up. Among 218 patients included in the study, 64 (29.4%) were diagnosed with DLBCL, 56 (25.7%) follicular lymphoma (FL), 31 (14.2%) CLL, 18 (8.2%) marginal zone lymphoma (MZL), 34 (15.6%) mantle cell lymphoma (MCL) and 15 (6.9%) with B-cell lymphoma unclassified.

### Cytological examination and FCI

For 218 patients included in our study, 192 lymph node fine needle aspiration biopsies (FNAB), 14 effusions, 7 cerebrospinal fluids, 1 bronchoalveolar lavages (BAL) and 4 peripheral blood samples were obtained for cytological examination and FCI. From each sample, 2 smears were prepared for microscopic examination and stained according to Giemsa and Papanicolaou method. Cells of the rest of the sample were suspended in cell media (4.5% bovine serum albumine, 0.45% EDTA in phosphate buffer solution with 50 IE/ml of penicillin) to prepare cell suspension for FCI. The absolute number of cells in total volume of cell suspension was determined in a Neubauer improved bright-line chamber. Samples for four-color FCI were prepared according to the protocol adopted for cytological samples at the Institute of Oncology, Ljubljana, Slovenia [12]. Antibodies by BD Biosciences were mostly applied, except in case of the antiCD52 antibody which was purchased from Serotec Ltd (Table 1). The samples were first filtered through 50 μm pore filter. Then, 200000 cells and 1.5 ml buffer (Cell Wash -BD Biosciences) were put in each test tube, mixed and centrifuged for 5 minutes at 1500 turns per minute. The supernatant was discarded and 3 or 5 μl of antibodies were added according to the antibody panel presented in Table 1. The samples were then mixed and incubated in dark for 20 minutes. After incubation, 1.5 ml buffer (Cell Wash - BD Biosciences) was added to the sample, mixed and centrifuged for 5 minutes at 1500 turns per minute. The supernatant was discarded and 300 μl of buffer (Cell Wash -BD Biosciences) was added [12]. The samples were measured with four-color flow-cytometer FACSCalibur (BD Biosciences). CellQuest software (BD Biosciences) was used for the acquisition and analysis of the results. In each test tube, we acquired 20 000 of CD45 or CD19 positive events depending on antibody panel presented in Table 1. For the analysis of the results, combined side scatter and surface marker (CD45 or CD19) gating was used.

**Table 1:** Standard antibody panel used in the diagnostics of B-cell lymphomas

	FITC		PE		PerCP/PerCP-Cy5.5		APC	
**Test tube**	**mAbs**	**Volume** **(μl)**	**mAbs**	**Volume** **(μl)**	**mAbs**	**Volume (μl)**	**mAbs**	**Volume** **(μl)**

1**	CD3*	5	CD19*	5	CD45*	5	CD20	3
2**	γ1-FITC	5	γ1-PE	5	CD19	5	γ1-APC	3
3**	Kappa*	5	Lambda*	5	CD19*	5	CD10	3
4**	FMC7*	5	CD23*	5	CD19*	5	CD5	3
5	CD52	3	CD11	5	CD19	5	CD38	3
6	CD4*	5	CD8*	5	CD3*	5	CD2	3

mAbs monoclonal antibodies, FITC fluorescein isothiocyanate, PE phycoerythrin, PerCP peridinin chlorophyll protein, PerCP-Cy5.5 peridinin chlorophyll protein-Cy5.5, APC-allophycocyanin, *BD Oncomark Reagents, ** when the samples were poorly cellular only test tubes 1-4 were applied

### Flow cytometric quantification of CD20 expression

For the quantification of the CD20 expression, the SPHERO Rainbow Calibration beads (Spherotech, Illinois, USA) were employed. The SPHERO Rainbow Calibration beads have been daily used in our laboratory for monitoring the performance status of flow-cytometer since 2001. Since these beads and the samples included in our study have been always acquired applying equal flow-cytometric settings, we could use the beads for the quantification of CD20 expression. The PMT Linearity QC Record software (Spherotech, Illinois, USA) was applied to determine the relative level of CD20 expression in MESF [13].

### Statistical analysis

Because the measurement variables did not meet the normality assumption of an anova, we used the non-parametric tests. Median, interquartile range and range between minimal and maximal variable were used for the description of CD20 expression. Kruskal Wallis and Mann Whitney tests were used to assess the difference in CD20 expression between the groups of patients with different histological types of B-cell lymphomas.

The research was approved by National Ethic Committee and was performed in compliance with the Helsinki declaration.

## Results

The CD20 expression in different B-cell lymphomas is presented in Table 2. The highest median CD20 expression was observed in the patients with DLBCL and the lowest in the patients with CLL. The range of CD20 expression in different B-cell lymphomas was wide, varying from 2 737 to 115 623 MESF in CLL and 3 549 to 679 577 MESF in DLBCL.

**Table 2:** CD20 expression in different histological types of B-cell lymphomas

Histological type of B-cell lymphoma (N)	CD20 expression (MESF)		
	Median	Range	IQR
DLBCL (64)	82 726	3 549 - 679 577	28 234 - 144 721
FL (56)	72 011	8 460 - 445 755	48 179 - 123 202
MCL (34)	66 375	8 826 - 423 799	39 725 - 99 915
MZL (18)	62 305	3 615 - 207 034	31 359 - 91 178
CLL (31)	14 064	2 737 - 115 623	8 275 - 26 855
NOS (15)	33 871	8 106 - 349 091	19 648 - 153 972

MESF...molecules of soluble fluorochrome, N...number of patients, IQR...interquartile range, DLBCL...diffuse large B-cell lymphoma, FL...follicular lymphoma, MCL...mantle cell lymphoma, MZL...marginal zone lymphoma, CLL...chronic lymphocytic leukemia, NOS...B-cell lymphoma unclassified

**Figure 1:**
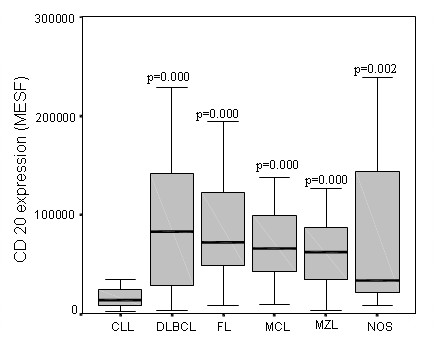
**CD20 expression in different B-cell lymphomas**. MESF....molecules of soluble fluorochrome, CLL...chronic lymphocytic leukemia, DLBCL...diffuse large B-cell lymphoma, FL...follicular lymphoma, MCL...mantle cell lymphoma, MZL...marginal zone lymphoma, NOS...B-cell lymphomas unclassified, NS...not significant.

When the CD20 expressions in DLBCL, FL, MCL, MZL, CLL and B-cell lymphomas unclassified were compared, it was found to be significantly lower (p = 0.002) only in CLL (Figure 1), but not significantly different from other lymphoma types (p = NS).

**Figure 2:**
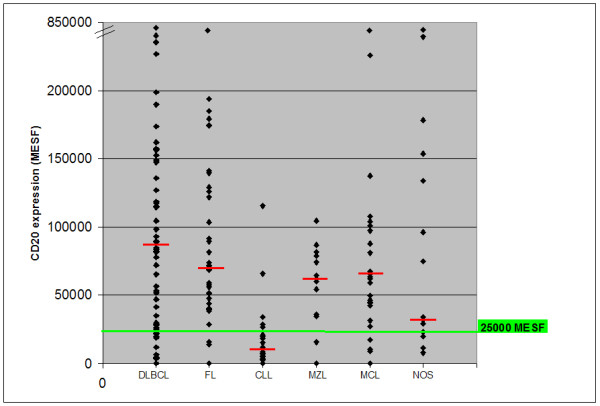
**CD 20 expression in B-cell lymphomas according to the cut-off value**. MESF...molecules of soluble fluorochrome, DLBCL...diffuse large B-cell lymphoma, FL...follicular lymphoma, CLL...chronic lympocytic leukemia, MCL...mantle cell lymphoma, MZL...marginal zone lymphoma, NOS...B- cell lymphoma unclassified, --... median values of CD 20 expression.

In our study population, 24.3% of patients had the level of CD20 expression below the cut-off value of 25 000 MESF. The highest percentage (74.2%) of patients with the level of CD20 expression below the cut-off value was noted in the group of patients with CLL while the lowest (7.1%) was observed in the group of patients with FL (Table 3, Figure 2). Table 3 also presents the number and the percentage of patients with the CD20 expression below the cut-off value in the present study compared to the results obtained in our previous study (8)

**Table 3:** Patients with the CD20 expression below the cut-off value

Histological type of B-cell lymphoma	Number of patients below the cut-off value/Total number of patients	% of patients below the cut-off value	Number of patients below the cut-off value/Total number of patients	% of patients below the cut-off value	Percentual difference in the proportion of patients below the cut-off value between the two studies
DLBCL	11/64	17.2	**6/42**	**14.3**	2.9
FL	4/56	7.1	**2/30**	**6.7**	0.4
CLL	23/31	74.2	**4/5**	**80.0**	5.8
MCL	6/34	17.6	**3/20**	**15.0**	2.6
MZL	4/18	22.2	**0/2**	**0**	22.2
NOS	5/15	33.3	**5/15**	**33.3**	0
Total	53/218	24.3	**20/114**	**17.5**	6.8

DLBCL...diffuse large B-cell lymphoma, FL...follicular lymphoma, CLL chronic lympocytic leukemia, MCL...mantle cell lymphoma, MZL...marginal zone lymphoma, NOS...B- cell lymphoma unclassified, **text in bold**...the results has been published in our previous study (M. Horvat et al. [8])

## Discussion

In the literature, there are only few data on the level of CD20 expression in different types of B-cell lymphoma. From the collected data, it may be concluded that the types of B-cell lymphoma differ from each other by CD20 expression; however, there is no clear evidence on the range of these differences and the variability of CD20 expression within each histological type of B-cell lymphoma.

Ginaldi et al. were the first to report that the level of CD20 expression in the patients with the leukemic form of B-cell lymphoma is significantly the lowest in CLL and the highest in hairy cell leukemia. No statistically significant difference was observed between the CD20 expressions in B-cell prolymphocytic leukemia, splenic MZL and MCL [3]. Similar results were reported by Huh et al. and Olejniczak et al. who compared the CD20 expressions in CLL, small B-cell lymphocytic lymphoma, acute lymphoblastic leukemia, hairy cell leukemia, DLBCL, FL, splenic MZL, and MCL [4,5]. Our results are consistent with those obtained by Ginaldi, Huh and Olejniczak, as they also show that the CD20 expression in the neoplastic cells of CLL is statistically significantly lower than that in other B-cell lymphomas included in our study (p = 0.002). We did not observe any statistically significant differences between the CD20 expressions in DLBCL, FL, MCL, MZL and B-cell lymphomas unclassified (p = NS). Though there was no statistically significant difference in the CD20 expressions in DLBCL, FL, MCL, MZL and B-cell lymphomas unclassified, the results of our study showed that the CD20 expressions varied significantly within different types of B-cell lymphomas. This may explain why the response to treatment with rituximab of the patients with the same type of lymphoma is so diverse.

Despite numerous clinical and preclinical studies on rituximab, the treatment with this drug has not outgrown its empirical use - we can confirm its efficacy only in the patients in whom the CD20 expression was determined in lymphoma cells, but we are not sure whether the level of surface CD20 expression may be a predictive factor, identifying the patients in whom the treatment with rituximab would be efficient and those in whom the use of this drug would be inefficient [14-16]. Results of two similar studies, investigating whether the level of CD20 expression is related to the response to treatment with rituximab, have been recently published. Johnson et al. have demonstrated that the CD20 expression and response to treatment with rituximab are connected. In the group of 272 patients with DLBCL treated with chemotherapy or immunotherapy with rituximab, the survival of patients with low CD20 expression was worse than that of patients with high CD20 expression [9]. The results of our most recent study are comparable to those of Johnson's study. In the study performed on 114 patients with different histological types of B-cell lymphoma, we found a correlation between the level of CD20 expression and response to treatment with rituximab. We also determined the cut-off value of CD20 expression at the level of 25 000 molecules of equivalent soluble fluorochrome (MESF) to be the predictor of response to rituximab containing treatment in the patients with B-cell lymphomas. This assumption was supported by our findings that the patients with the level of CD20 expression above the cut-off value had a significantly longer overall survival (p = 0.038) and a significantly higher overall response rate (p < 0.001) than the patients with the level of CD20 expression below the cut-off value. Yet, we observed no significant difference in the response duration between the two groups. By means of the determined cut-off level of CD20 expression we estimated that approximately one fifth of the B-cell lymphoma patients with low CD20 expression will not respond to rituximab containing treatment at all or will not respond optimally [8].

Because this presumption was based on the results of the study including only a smaller series of patients, the proportion of patients with the CD20 expression below the cut-off value might not be necessarily representative. Namely, in the reported study, some B-cell lymphoma groups consisted of only few patients, for example of only 2 patients in the MZL group and of only 5 patients in the CLL group. The reliability of the proportion of patients having the CD20 expression below the cut-off value in these two groups might therefore be questionable.

Having expanded the study population, we detected that almost 25% of B-cell lymphoma patients had the CD20 expression below the cut-off value of 25 000 MESF. This proportion is higher than the 17.5% reported in our previous study. Among the patients with the CD20 expression below the cut-off value, we found prevailingly the patients with CLL (74.2%) and most infrequently the patients with FL (7.1%). This distribution of low CD20 expression is for the most part in concordance with our previous findings [8]. However, the proportion of patients with the CD20 expression below the cut-off value varied in different B-cell lymphoma groups when we expanded the study population. It increased substantially in MZL (for as much as 22.2%) and decreased in CLL patient group (for 5.8%). In the groups of patients with DLBCL, FL and MCL, on the other hand, we observed only a slight increase in the percentage of patients having the CD20 expression below the cut-off limit - the increase being between 0.4 to 2.9%. We believe, that these changes are attributable to the altered number of patients belonging to each of the B-cell lymphoma groups.

The results of our study indicate that the level of CD20 expression is an important predictive factor for the response to rituximab containing treatment. Still, we believe that further studies on the intensity of CD20 expression should be carried out in a homogeneous population of patients with identical histological types of B-cell lymphoma.

## Conclusions

CD20 expression in CLL is significantly lower than in most histological types of mature B-cell lymphomas in which it appears to be very comparable. Almost 25% of B-cell lymphoma patients have the CD20 expression below the cut-off value of 25 000 MESF which was proposed to be the predictor of response to treatment with rituximab. This proportion (24.3%) is higher than the one reported in our previous study (17.5%). Further studies are needed for the patients with every histological type of B-cell lymphoma to confirm our observations that the patients with low CD20 expression respond to rituximab containing treatment poorly or not at all.

## Competing interests

The authors declare that they have no competing interests.

## Authors' contributions

VKP designed the study and wrote the manuscript. JL carried out the flow cytometric data acquisition and analysis. MH performed statistical analysis and assisted in flow cytometric data analysis. BJN participated in clinical discussion and reviewed the manuscript. All authors read and approved the final manuscript.
